# Scientometric Study on Non-communicable Diseases in Iran: A Review Article

**Published:** 2018-07

**Authors:** Niloofar PEYKARI, Hassan HASHEMI, Gholamreza ASGHARI, Mohammadhadi AYAZI, Ghasem JANBABAEI, Reza MALEKZADEH, Alireza RAEISI, Ali SADROLSADAT, Mohsen ASADI-LARI, Aliasghar FARSHAD, Farshad FARZADFAR, Mostafa GHANEI, Ali Akbar HAGHDOOST, Ramin HESHMAT, Hamidreza JAMSHIDI, Afshin OSTOVAR, Amirhossein TAKIAN, Bagher LARIJANI

**Affiliations:** 1. Iranian Non Communicable Diseases Committee (INCDC), Ministry of Health and Medical Education, Tehran, Iran; 2. Food and Drug Organization, INCDC, Ministry of Health and Medical Education, Tehran, Iran; 3. Social Health Deputy, INCDC, Ministry of Health and Medical Education, Tehran, Iran; 4. Curative Affairs Deputy, INCDC, Ministry of Health and Medical Education, Tehran, Iran; 5. Research and Technology Deputy, INCDC, Ministry of Health and Medical Education, Tehran, Iran; 6. Public Health Deputy, INCDC, Ministry of Health and Medical Education, Tehran, Iran; 7. Development Deputy, Management, and Resources, INCDC, Ministry of Health and Medical Education, Tehran, Iran; 8. International Affairs, INCDC, Ministry of Health and Medical Education, Tehran, Iran; 9. Occupational Health Research Center, Iran University of Medical Sciences, Tehran, Iran; 10. Non Communicable Diseases Research Center, Endocrinology and Metabolism Research Institute, Tehran University of Medical Sciences, Tehran, Iran; 11. Chemical Injuries Research Center, Baqiyatallah University of Medical Sciences, Tehran, Iran; 12. Planning and Coordination Deputy, INCDC, Ministry of Health and Medical Education, Tehran, Iran; 13. Chronic Diseases Research Center, Endocrinology and Metabolism Research Institute, Tehran University of Medical Sciences, Tehran, Iran; 14. Dept. of Pharmacology, School of Medicine, Shahid Beheshti University of Medical Sciences, Tehran, Iran; 15. Osteoporosis Research Center, Endocrinology and Metabolism Research Institute, Tehran University of Medical Sciences, Tehran, Iran; 16. Health Equity Research Center, Tehran University of Medical Sciences, Tehran, Iran

**Keywords:** Non-communicable diseases, Scientometry, Iran

## Abstract

**Background:**

Non-Communicable disease (NCDs) is a killer of people that needs to urgent actions across the world. Scientific evidence is the critical arm for effective interventions. Therefore, we aimed to quantify the trend of four main NCDs’ scientific publication in a 17-yr period, and reflect international collaboration.

**Methods:**

This scientometric study on four main NCDs; cardiovascular diseases, cancers, diabetes, and chronic respiratory diseases were carried out through the narrative review in international databases of Scopus from 2000 to 2016. In this way, the number of articles, citations, and international collaboration were assessed, and the frequently used terms on noncommunicable diseases were mapped by VOSviewer software.

**Results:**

Over the 17 years, 25827 articles about four main NCDs by Iran indexed in Scopus have increasing trend steadily. However, chronic obstructive respiratory publications have slow trend. The number of articles, citations, and h index of cancer-related publications was higher than the others. Diabetes, cardiovascular diseases, and chronic respiratory diseases scientometrics indicators state in next positions, respectively. The most collaborative country was USA in the four areas, and there was not seen region countries’ collaboration in top ten levels. The frequently used terms in NCDs’ articles in order were diabetes, cardiovascular diseases, and breast cancer.

**Conclusion:**

Iran provides appropriate face of cancer, diabetes, and cardiovascular diseases publications in the mirror of NCDs’ scientometry. However, there is need for more effort in chronic respiratory diseases researches, and strengthen collaboration with regional countries.

## Introduction

In 2016, Non-communicable diseases (NCDs) killed 287 thousand people in Iran. Ascending trends of the number of related death and disability-adjusted life years (DALYs) during the past decades show the terrific threat for Iran. NCDs not only result in premature death but also lead to considerable disability (1, 2). Occurrence 6.5 million years of life lost (YLLs), and 8.2 million years lived by disability (YLDs) is the dramatic sign of NCDs emerging (3). Therefore, Iran same as other countries is responsible to prevention and control NCDs and response to call of WHO for 25% reduction by 2025 in premature mortality from non-communicable diseases (4, 5).

In this way, Iran developed NCDs’ national action plan for prevention and control NCDs through inter and intra-sectoral collaboration and motivate policymakers in this responsibility (4). Through this action plan, appropriate activities could be performed, but effective interventions need the scientific evidence and eliminate the gap between policymakers and researchers. In each country, the knowledge production is responsibility of researchers to present reliable evidence for policymakers (6).

Scientometric study is the mirror of produced knowledge and reflects gaps in each area (7). Authorship and citation are indicator of research activity and reputation of evidence. In addition, collaboration measurement is an indicator of research systems’ structure at the macro level (7).

The existence of dramatic problem of NCDs and the role of scientometric study lead us to present study. Among non-communicable diseases, cardiovascular diseases, cancers, diabetes, and chronic respiratory diseases are the four main NCDs. About 82% of deaths occurred because of these main diseases (3, 5). Through this scientometric study, we assessed the trends of published articles and citations in the four main NCDs in Iran from 2000 to 2016.

## Methods

This scientometric analysis focused on four main non-communicable diseases; cardiovascular diseases, cancers, diabetes, and chronic respiratory diseases researches in Iran, from 2000 to 2016. As the wide coverage of Scopus database in health and biomedicine disciplines and presentation the valid citation reports of knowledge products, we search this international database (8).

We developed search strategy and it validated by external scientific group and in this way, we retrieved all related records (Table 1). We had limitation on time (2000–2016), and Iran based on authors’ affiliation or address.

**Table 1:** Search strategy utilized in this study

***Four Main NCDs***	***Search strategy***
Cardiovascular Diseases	(TITLE-ABS-KEY (“Cardiovascular Diseases” OR “Cardiovascular Disease” OR “Ischemic heart disease” OR “Ischemic heart diseases” OR “Coronary artery diseases” OR “Coronary artery disease”) AND AFFIL (Iran OR I.R.Iran)) AND PUBYEAR > 1999 AND PUBYEAR < 2017
Cancers	(TITLE-ABS-KEY (Neoplasms OR Neoplasm OR Neoplasia OR Neoplasias OR Malignancy OR Malignancies OR Cancer OR Cancers) AND AFFIL (Iran OR I.R.Iran)) AND PUBYEAR > 1999 AND PUBYEAR < 2017
Diabetes	(TITLE-ABS-KEY (“Diabetes Mellitus” OR diabetes) AND Iran[Affiliation] AND AFFIL (Iran OR I.R.Iran)) AND PUBYEAR > 1999 AND PUBYEAR < 2017
Chronic Respiratory Diseases	(TITLE-ABS-KEY (“Chronic Respiratory Diseases” OR “Chronic Respiratory Disease” OR “Pulmonary Disease, Chronic Obstructive” OR copd OR “Chronic Obstructive Pulmonary Disease” OR coad OR “Chronic Obstructive Airway Disease” OR “Chronic Obstructive Airway Diseases”) OR TITLE-ABS-KEY (“Chronic Obstructive Lung Disease” OR “Chronic Obstructive Lung Diseases” OR “Airflow Obstruction” OR “Airflow Obstructions” OR “Chronic Airflow Obstructions” OR “Chronic Airflow Obstruction”) AND AFFIL (Iran OR I.R.Iran)) AND PUBYEAR > 1999 AND PUBYEAR < 2017

All results stored in EndNote X7 software, Thomson Reuters, USA. Because of some overlap in databases, duplicate cases were extracted and excluded. By systematic search in Scopus, the number of publications, citation, and h index as the main indicators in scientometrics retrieved and analyzed. H index reflects the most cited papers and the number of citations. The definition of the h index is that “a scholar with an index of h has published h papers each of cited in other papers at least h times” (9). In this way, the source of publications, document type, and responsible institutes, and collaborative countries were assessed.

For assessing the position of considered diseases among NCDs, we systematically searched the terms of non-communicable, Chronic Disease, Chronic Illness, and Chronically Ill in Scopus and mapped it in VOSviewer software.

We used VOSviewer mapping software of Centre for Science and Technology Studies, Leiden University, The Netherlands (10). By using this software and consider thresholds of minimally 10 fractionally counted papers for each term; a national map of NCDs’ articles was generated. For each term meet the threshold, a relevance score calculated and based on this score, the most relevant terms were selected. For mapping results, maps created based on title and abstract field.

## Results

### Trend of Four Main Non-communicable Diseases Related Publications in Iran

**Fig. 1:**
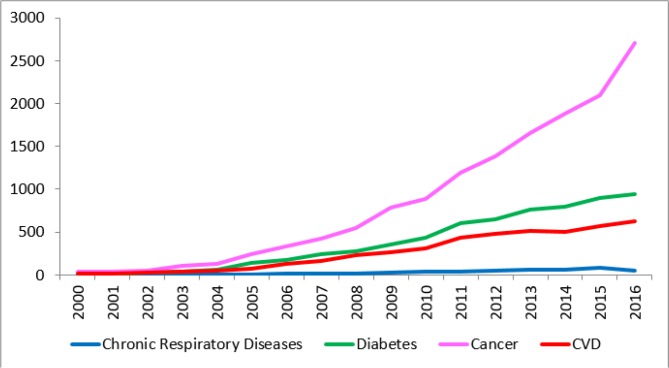
The trend of publications on Four Main NCDs in Iran from 2000 to 2016

During the past 17 years ago, 25827 articles published about cardiovascular diseases, cancers, diabetes, and chronic respiratory diseases by Iran indexed in Scopus. The most articles were related to cancer (n=14500) and the number of publications about Diabetes, and CVDs was 6426, and 4417. The fewer publications number in this period was related to chronic respiratory diseases (n=484). The time trend of these articles has been shown in Fig. 1.

Published articles about cancers had sharp ascending trend, but chronic respiratory diseases related articles had very low slope. The time trend of CVDs’ publications was lower than diabetes’ articles. Parallel with ascending trends of considered publications, their citation has to increase by the same pattern. The citations and h-index in four area are as follow; Cardiovascular Diseases (49358, 77), Cancer (124209, 105), Diabetes (68050, 87), and Chronic respiratory diseases (8979, 36).

### The Four Main Non-communicable Diseases Articles Type and their sources

**Fig. 2:**
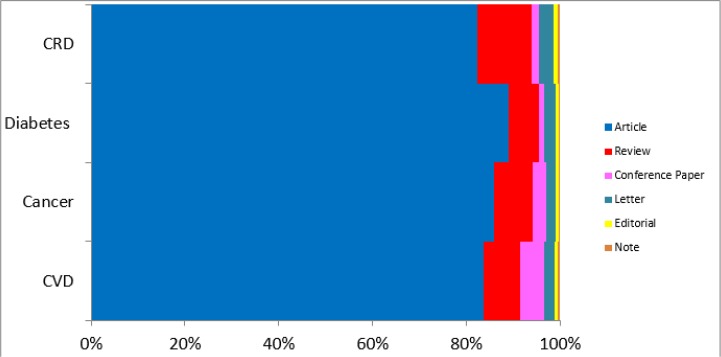
The pattern of four main Non-communicable Diseases’ article types based on Scopus database

Based on Scopus database, more than 80% of published articles related to CVDs, Cancer, Diabetes, and Chronic respiratory diseases were original articles. About 8% of these publications were review articles, and the remained publications were editorial, letter, note, and conference papers (Fig. 2).

In ranking the source of publications revealed that the most articles during 17 years ago published in following journal according to related area. Journal of Tehran University Heart Center (131 article) in CVDs, Asian Pacific Journal of Cancer Prevention (879 article) in Cancer, Iranian Journal of Endocrinology and Metabolism (172 article) in diabetes, and Tanaffos (57 article) in Chronic respiratory diseases were the top rank journals in considered publications.

### Authorship and International collaboration among Four Main Non-communicable Diseases Articles

Authorship the most articles by subject of the four main NCDs allied to Tehran University of Medical Sciences. Researchers of Shahid Beheshti University of Medical Sciences were the second authors of these articles.

In cardiovascular diseases and Diabetes area, Isfahan University of Medical Sciences state in the third rank of authorship, but in Cancer, Shiraz University of Medical Sciences, and in chronic respiratory diseases subject, Baghiatallah University of Medical Sciences had the third rank. Based on Scopus’ results from 2000 to 2016, the most collaboration in four main noncommunicable Diseases’ articles was related to United State. In Table 2, top ten collaborative countries in four areas presented.

**Table 2:** Top ten collaborative countries in four Main Non-communicable Diseases Articles

***Cardiovascular Diseases***		***Cancers***		***Diabetes***		***Chronic Respiratory Diseases***	
***Countries***	***Publications(N)***	***Countries***	***Publications(N)***	***Countries***	***Publications(N)***	***Countries***	***Publications(N)***
United States	294	United States	893	United States	275	United States	47
United Kingdom	223	United Kingdom	339	United Kingdom	149	Netherlands	44
Australia	108	Canada	322	Canada	135	United Kingdom	32
Canada	107	Germany	291	Australia	117	Canada	25
Netherlands	99	Sweden	250	Italy	63	Italy	24
Italy	68	Italy	245	Malaysia	63	Australia	20
India	65	Australia	204	India	56	France	17
Malaysia	63	France	178	Sweden	56	Brazil	12
Sweden	60	Malaysia	175	Netherlands	45	Germany	12
France	58	Netherlands	138	Germany	39	Sweden	12

**Fig. 3:**
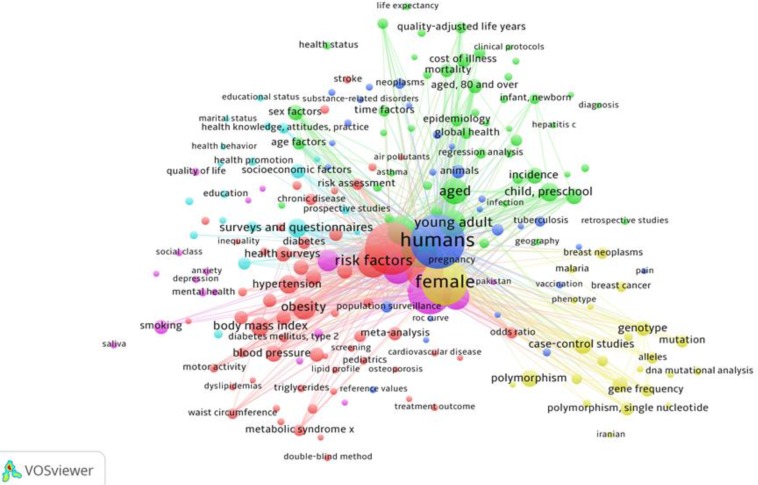
Map of frequent terms in Iranian Non-communicable Diseases articles indexed in Scopus

In mapping the non-communicable diseases search results, from the 10294 terms, 517 terms meet the threshold. According to relevance score, 327 terms selected as relevant terms. Figure 3 shows the map of connection lines between frequent terms in Iranian Non-communicable Diseases articles

In this map, each subject area demonstrated in colored regions. In addition, the bubbles’ size and related font size is an indication of terms frequency. The same colors show the terms relation clusters. The bubble of diabetes is larger than cardiovascular diseases, and among neoplasm, breast cancer appears on this map. Chronic respiratory diseases are invisible between bubbles.

## Discussion

Alarming increase of non-communicable diseases burden needs to pay attention to all policies (4, 5, 11, 12). Scientific evidence are essential for Justification of the need for implementation effective intervention for prevention and control NCDs. Therefore, the researchers attend to knowledge production and dissemination across the world (13).

According to our study, literature about cancers, diabetes, and cardiovascular diseases significantly had ascending trend, but chronic respiratory diseases had slow increase during the 17 yr. The other scientometric study showed CVDs is interesting subject for researchers, and in the ten years period, the number of CVDs’ publication had three times increase (14).

In addition, increasing trend of cancers’ publication in other countries revealed the increasing interest in this area (15). In addition to present study, the others emphasize on ascending trend of diabetes publication in Iran, and turkey (16, 17).

However, the trend of chronic respiratory diseases publication had different pattern in different country notwithstanding are in the same region. A scientometric study revealed that Iranian scientific publications are not related to burden of diseases (18, 19). By attention to sharp ascending trend of COPD’s death in Iran, it is better the researchers of this area pay more attention to produce scientific evidence for implementing appropriate interventions. Almost the number of publications depends on related research centers activities.

Despite the need for evidence-based decision-making and the value of review articles in evidence, pattern of article types showed only 8% of publications state in this category. Encourage researchers to more attention to review articles could be beneficial for us (20–22). Exchange the experiences, and knowledge transition in national and international levels could be useful in establishment collaborative network. The result of international collaboration analysis demonstrated the view of joint project between countries. There is not strong collaboration between our region’s countries, while similarity of conditions among Middle East countries should be noticeable for scientific collaboration (16). Some regions have strong collaboration in research such as North America and west Europe, (14) but some country such as China could be more attention in this area (23).

The scientometrics map on non-communicable diseases shows the small size of cardiovascular diseases bubble compares with diabetes bubble. It is related to research centers activities (24). The other point is the hole of chronic respiratory diseases bubble in this map. This is the other sign of needs to more attention to produce scientific evidence in this area. Assess the frequent terms used in Iranian articles about non-communicable diseases revealed that the terms of “human,” “female”, and “risk factors” are frequent terms in Scopus database. Frequently used of “females” may be sign of more participation of women in biomedical researches.

Our study has some strength points. First, we used Scopus database by the most coverage in health and biomedical publications, and the Second, our study is the rare scientometric study by considering four main NCDs (16, 24–26). However, we faced some limitation such as; presence multidisciplinary subject category and a little overlap between some areas that considered in scientometrics analysis by VOSviewer software.

## Conclusion

This study provides a scientometrics analysis about non-communicable researches in Iran. Ascending trend of cancer and diabetes publication is appreciated but there is need to encourage researchers to produce more evidence about cardiovascular diseases and chronic respiratory diseases for evidence-based decision-making. Moreover, create networking in the region could help in knowledge exchange, and at the end we hope for evidence-based policy making in this important area that kills our communities and wastes our resources.

## Ethical considerations

Ethical issues (Including plagiarism, informed consent, misconduct, data fabrication and/or falsification, double publication and/or submission, redundancy, etc.) have been completely observed by the authors.
